# Following lithiation fronts in paramagnetic electrodes with *in situ* magnetic resonance spectroscopic imaging

**DOI:** 10.1038/ncomms13284

**Published:** 2016-11-03

**Authors:** Mingxue Tang, Vincent Sarou-Kanian, Philippe Melin, Jean-Bernard Leriche, Michel Ménétrier, Jean-Marie Tarascon, Michaël Deschamps, Elodie Salager

**Affiliations:** 1 CNRS, CEMHTI UPR3079, Université d'Orléans, 1D avenue de la recherche scientifique, 45071 Orléans Cedex 2, France; 2 Réseau sur le Stockage Electrochimique de l'Energie (RS2E), CNRS FR3459, 33 rue Saint Leu, 80039 Amiens Cedex, France; 3 Laboratoire de Réactivité et de Chimie des Solides (UMR 7314), Université de Picardie Jules Verne, 33 rue Saint Leu, 80039 Amiens Cedex, France; 4 ICMCB, CNRS UPR9048, Université de Bordeaux, ENSCBP, 87 avenue du Dr A. Schweitzer, 33608 Pessac Cedex, France; 5 Collège de France, CNRS FRE3357, 11 place Marcelin Berthelot, 75005 Paris, France; 6 Alistore European Research Institute, CNRS FR3104, 33 rue Saint Leu, 80039 Amiens Cedex, France

## Abstract

Li-ion batteries are invaluable for portable electronics and vehicle electrification. A better knowledge of compositional variations within the electrodes during battery operation is, however, still needed to keep improving their performance. Although essential in the medical field, magnetic resonance imaging of solid paramagnetic battery materials is challenging due to the short lifetime of their signals. Here we develop the scanning image-selected *in situ* spectroscopy approach, using the strongest commercially available magnetic field gradient. We demonstrate the ^7^Li magnetic resonance spectroscopic image of a 5 mm-diameter operating battery with a resolution of 100 μm. The time-resolved image-spectra enable the visualization *in situ* of the displacement of lithiation fronts inside thick paramagnetic electrodes during battery operation. Such observations are critical to identify the key limiting parameters for high-capacity and fast-cycling batteries. This non-invasive technique also offers opportunities to study devices containing paramagnetic materials while operating.

## Abstract (web summary)

Magnetic resonance imaging is a promising non-invasive approach to visualize paramagnetic materials in devices, but the short lifetime of signals currently limits its use. Here, the authors develop an approach which overcomes this hurdle to spectroscopically image lithiation fronts during battery operation.

Our planet faces formidable sustainability challenges that call for rigorous research in various disciplines including, among others, the field of electrochemical energy storage. Batteries are today essential in tackling global warming and energy security, but sustaining such a mission calls for new advances in battery performance. To better understand the remaining limitations, notably in terms of charging rates and capacity, analytical techniques must be pushed to their limits to characterize *in situ*, in a non-invasive and non-destructive way, the internal parts of batteries in operating conditions.

While thick electrodes (several hundreds of micrometres) are a promising route to increase capacity in batteries^1^, diffusion in the electrode and hence electrode processing appears to be the limiting step to maintain suitable charging rates. Visualizing the distribution of lithium ions inside an electrode during operation (*operando*), however, remains a difficult task. So far, many *in situ* imaging techniques have been developed to follow the evolution of electrode materials upon cycling^2^,^3^, using neutron or synchrotron sources^4^,^5^,^6^,^7^,^8^,^9^,^10^,^11^,^12^,^13^,^14^ and nanoscale microscopy^15^,^16^,^17^,^18^. Scattering or spectroscopic techniques generally provide contrast on these images through elemental content, oxidation state or crystalline phase mapping. Nuclear magnetic resonance (NMR)—a non-invasive and highly lithium-sensitive bulk technique—combines imaging and spectroscopy and it is, therefore, an insightful and complementary tool. The relevance of NMR has been proven to investigate *in situ* charging and discharging of batteries in realistic conditions, so that *operando* characterization of lithium-ion batteries by ^7^Li magnetic resonance spectroscopy or imaging is currently explored enthusiastically^19^,^20^,^21^,^22^,^23^,^24^,^25^,^26^,^27^,^28^,^29^,^30^,^31^. So far, however, no magnetic resonance spectroscopic imaging, that is, data correlating the NMR spectrum with the spatial location inside the battery, has been reported for battery electrodes containing paramagnetic compounds, to the best of our knowledge. A first attempt to image LiFePO_4_ with stray-field NMR was recently reported, but the image was distorted by the strong paramagnetism and no spectroscopic information could be obtained^24^. Standard spectroscopic imaging methods were nicely exploited to visualize *in situ* the battery components with a sharp NMR signal: lithium dendrites^20^,^21^,^30^ or liquid electrolyte in batteries^25^ and supercapacitors^32^. The paramagnetic electrodes are always absent from these images due to the extremely short lifetime of their NMR signal.

In the classical methodology for spectroscopic imaging (chemical shift imaging (CSI)^33^) or localized spectroscopy (volume selective, using spin/stimulated echoes^34^,^35^,^36^), intense pulsed magnetic field gradients (PFG) are applied to spatially label transverse magnetization. Switching the gradient off is necessary to record the high-resolution NMR spectrum and cannot be completed faster than in 1 ms for large PFG strengths. Due to the short transverse relaxation time (*T*_*2*_') induced by paramagnetism of the transition metal atoms, even for the nearby lithium atoms, the NMR signal in the electrodes decays quickly (typically <100 μs). As a consequence, classical NMR spectroscopic imaging techniques are not suitable to detect the electrode materials in the image^25^, even though they perform well for the liquid electrolyte^32^. Complementary approaches for imaging solid materials with ‘ultra-short *T*_2_' (refs 37, 38, 39, 40, 41) have been developed but none of them enable the NMR spectrum and image to be jointly recorded. Consequently *in situ* spectroscopic imaging of today's most praised paramagnetic electrodes such as LiFePO_4_ or Li-rich NMC (Li(Li_*x*_M_1-*x*_)O_2_, M=Mn, Co, Ni) was deemed impossible.

Here we tackle this long-lasting challenge in the hope of bringing additional tools to improve battery performance. To circumvent the limitation in transverse relaxation time, we develop a strategy taking advantage of the considerably longer longitudinal relaxation time (*T*_*1*_>5 ms) for paramagnetic materials compared with their transverse relaxation time. This concept was proposed by Ordidge *et al*. in 1986 (ref. 42) as the ISIS sequence (for image-selected *in vivo* spectroscopy) to get localized ^31^P NMR spectra as a complement to standard ^1^H MRI (magnetic resonance imaging) *in vivo* images, but it was never implemented within the battery community nor exploited for imaging purposes. We develop an approach denoted scanning image-selected *in situ* spectroscopy (S-ISIS) to fully image a 5 mm-diameter operating battery with a resolution of 100 μm. Such resolution for paramagnetic materials can only be achieved by taking advantage of the strongest commercially available pulsed magnetic field gradient (30 T m^−1^). Time-resolved S-ISIS images enable the visualization *in situ* of the displacement of lithiation fronts inside thick paramagnetic electrodes during battery operation. This non-invasive tool, based on the combination of spatial and spectroscopic information, enables the diagnosis of the limiting steps in battery performance. While our results are limited to one-dimension due to the lack of strong three-dimensional pulsed field gradient with short switching times, they are directly transposable to three-dimensional if suitable three-dimensional gradients are developed.

## Results

### Standard ISIS approach

**Figure 1:**
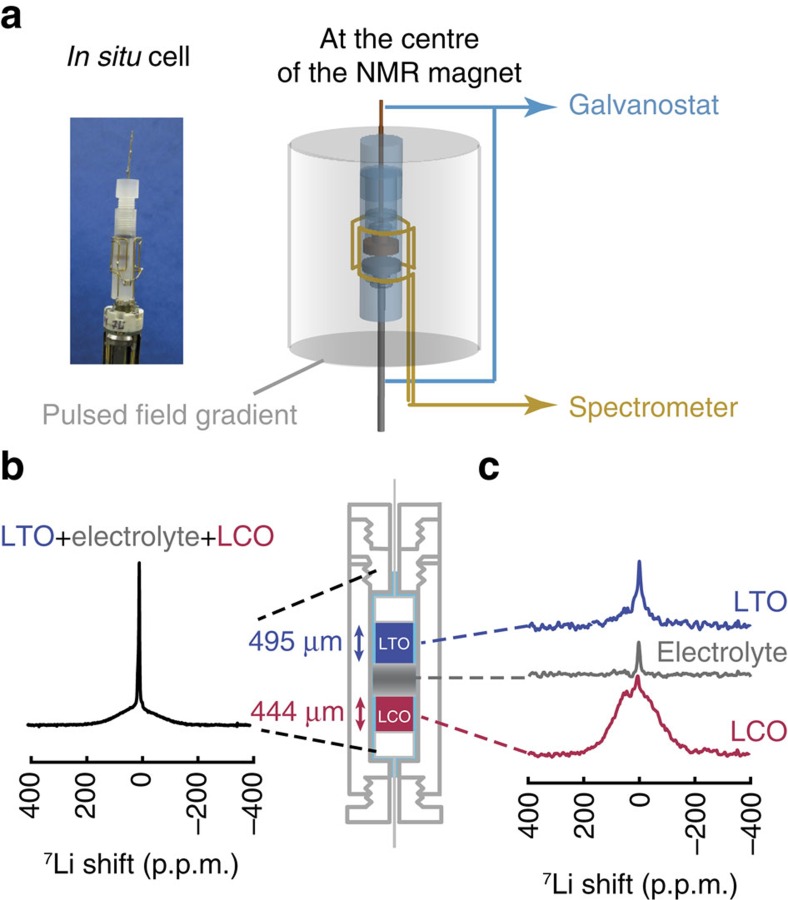
Experimental set-up and localized NMR spectra. (**a**) The *in situ* electrochemical cell is located in the centre of the radio-frequency and PFG coils used for spectroscopy and imaging; it is connected to a galvanostat for (dis)charging. (**b**) 1D ^7^Li NMR spectrum showing the overlap of the signals of the two electrodes (LCO, LTO) and of the liquid electrolyte. The electrolyte is soaking the whole cell. (**c**) Localized spectra obtained with ISIS for LTO, the electrolyte in the separator and LCO. The localized spectra of the electrodes contain a sharp component at 0 p.p.m. due to Li^+^ ions in the liquid electrolyte soaking the electrodes. The electrolyte peak is more intense in **b** because of contributions from the liquid at the top and bottom of the electrodes (not shown in **c**).

We use the cylindrical electrochemical cell (Fig. 1a) that we developed earlier for *operando* NMR^25^. Two composite electrodes with active materials LiCoO_2_ (LCO, 444 μm-thick) and Li_4_Ti_5_O_12_ (LTO, 495 μm-thick) for the positive and negative electrodes, respectively, are assembled with a separator soaked in a commercial electrolyte containing Li^+^ ions (details in Methods). The electrodes are made deliberately as thick (around 500 μm) and dense pellets (SEM micrographs in Supplementary Fig. 1) for which transport and kinetic limitations are critical. The knowledge acquired from the understanding of limitations in thick electrodes is also expected to shed light on the capacity loss in commercial thinner electrodes (usually 100 μm-thick), when charged at very fast regimes.

The ^7^Li Hahn-echo spectrum of the full electrochemical cell is shown in Fig. 1b; note that the signals from Li atoms in the two electrodes and in the liquid electrolyte are overlapping. At first sight, this suggests that monitoring compositional changes of Li in each electrode upon cycling might be precluded. However, as shown in Fig. 1c, the standard ISIS approach succeeds in recording ^7^Li spectra for each battery element (positive electrode, negative electrode or electrolyte adsorbed in the separator). We demonstrate in the following that this approach works even in the least favourable case of the charged battery (paramagnetic Li_*x*_CoO_2_ and Li_*y*_Ti_5_O_12_, *x*=0.5 and *y*=6.6).

### Scanning-ISIS of a full battery

As a second step, we push further the technique with the development of scanning-ISIS (S-ISIS) to provide a full spectroscopic image of lithium within the battery. The S-ISIS imaging technique consists in scanning successive slices in the whole battery; the Li one-dimensional spectroscopic image is reconstructed by stacking the spectra obtained for each slice (see SI for a detailed explanation).

**Figure 2:**
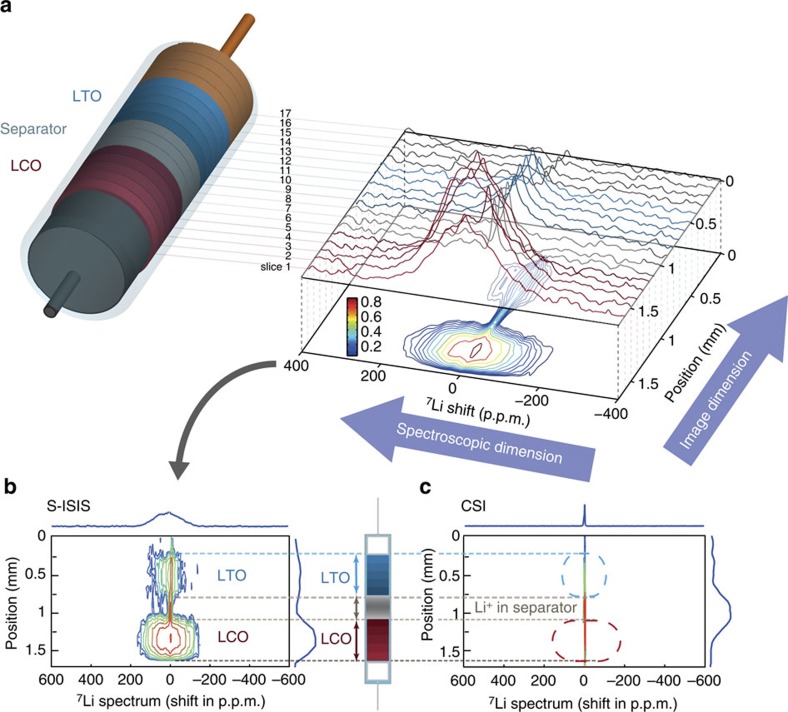
^7^Li Scanning ISIS of the LCO/LTO battery. (**a**) Series of spectra obtained for horizontal slices of thickness 100 μm in the battery. Slices 1, 5 and 13 are less intense because they are astride the electrode and the electrolyte. The spectroscopic image is reconstructed from the series of spectra and correlates spectrum and position. The corresponding contour map is shown below. Signal intensity for each contour in the map is colour-coded as indicated by the colour bar. The electrolyte soaking the whole cell is evidenced in all slices by a sharper signal close to 0 p.p.m., overlapping with the electrode spectrum. (**b**,**c**) Comparison of the NMR spectroscopic images for the LCO/LTO battery obtained with (**b**) the S-ISIS method (FOV 1.7 mm, 17 slices, no slice gap) and (**c**) the standard CSI (FOV 2.5 mm, only 1.7 mm shown, that is, 27 slices). No signal is obtained for LTO and LCO in the CSI spectroscopic image, contrary to the S-ISIS image. Spectral width: 200 kHz; acquisition time: 3 h 20 min for S-ISIS, 3 h 38 min for CSI; resolution: 100 μm for S-ISIS, 77 μm for CSI.

Figure 2a presents the 17 stacked spectra for an assembled LCO/LTO battery before cycling. A more elegant way to visualize the S-ISIS image is to reconstruct the spectroscopic map and display it as a contour map (below the stacked spectra in Fig. 2a,b), which correlates the position in the battery (image dimension) with the ^7^Li spectrum (spectroscopic dimension).

This image, with a field of view of 1.7 mm, was acquired in 3 h 20 min using 17 successive slices of 100 μm with no slice gap. Slice resolution for the electrodes depends notably on the NMR homogeneous linewidth (see SI for details), which is strongly related to their oxidation state. Resolution may, therefore, vary across the battery and when (dis)charging. Based on the strongest broadening—in that case LTO on top of charge, we chose to limit our resolution to 100 μm for all slices at all states-of-charge, to ensure a near-constant resolution. Contrary to the classical CSI sequence (Fig. 2c) for which the liquid electrolyte is the only Li-bearing species detected, the S-ISIS spectroscopic image contains spectra for the solid electrodes containing lithium ions (with a narrow contribution near 0 p.p.m. for the electrolyte soaking the electrode). All Li-containing parts of the battery are visible in S-ISIS (from top to bottom): electrolyte drops trapped near the Cu current collector (slices 17 to 14), the LTO composite electrode (slices 13 to 8), the electrolyte in the separator (slices 8 to 5) and LCO (slices 5 to 1). Slice 8 and slice 5 contain signal from the electrolyte and the LTO electrode and from the electrolyte and the LCO electrode, respectively (see Supplementary Fig. 2). The ^7^Li signal of the slice containing the separator is narrow as expected for Li^+^ ions in the electrolyte, with slight variations on the peak position due to disparities in the magnetic susceptibility along the battery. Note that ample intensity is obtained with S-ISIS despite probing only 40 and 80 μmol of Li per slice in LCO and LTO, respectively.

The correlation of image and spectroscopic information brings a powerful differentiating factor (contrast) for the electrodes; the peaks in the spectra are all centred at 0 p.p.m. in the initial (uncharged) state of the battery because LiCoO_2_ and Li_4_Ti_5_O_12_ are diamagnetic, but their widths are clearly different when moving along the battery. As shown by the colour bar on the left, the red contour corresponds to the highest signal intensity and the blue contour to the lowest signal intensity. The spectrum of LCO is broadened by the ^7^Li-^7^Li dipolar couplings and the contribution of paramagnetic defects^43^, while LTO is relatively narrow as expected for a well-crystallized diamagnetic material. The signal for LTO is weaker than for LCO (no red 0.9 contour line) due to a longitudinal relaxation (*T*_*1*_) filtering effect in the pristine state.

### *In situ* monitoring of the full battery with S-ISIS

**Figure 3:**
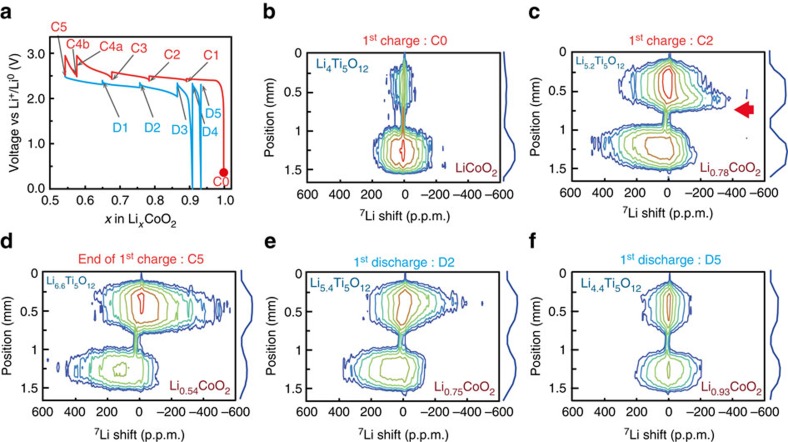
*In situ* S-ISIS spectroscopic images of the LCO/LTO battery. (**a**) Electrochemical profile of the *in situ* battery cycled so that Li_0.5_CoO_2_ is reached theoretically in 25 h (C/25). The starting point (pristine) is marked as a red dot. The vertical lines are due to voltage relaxation before the acquisition of the images. (**b**–**d**) Images recorded upon charge, corresponding to pristine (C0), *x*=0.78 (C2) and *x*=0.54 (C5). (**e**) Battery at half-discharge (*x*=0.75) and (**f**) after one cycle (*x*=0.93). The colour code of the contours is the same in all plots.

The protocol for recording a single image being defined, we extend our approach to monitor *in situ* the battery during operation. Figure 3a shows the evolution of the voltage of the battery as a function of the state of charge (SOC), indicated here by the number of Li per Co, *x* in Li_*x*_CoO_2_. Note that as the battery is charged, Li atoms move out of LiCoO_2_ and into Li_4_Ti_5_O_12_, resulting in oxidation of Co^(III)^ to paramagnetic Co^(IV)^ in the positive electrode and reduction of Ti^(IV)^ to paramagnetic Ti^(III)^ in the negative electrode. The battery was charged in five steps and S-ISIS images were acquired in 2h45 after letting the battery rest at the given SOC in open-circuit mode for 7 h (details in Methods and Supplementary Fig. 3). These open-circuit times correspond to the vertical lines in Fig. 3a, during which the voltage changes but not *x* due to the disappearance of the (dis)charge-generated overpotential. The effect is much stronger at the end of charge and discharge as we reach the limits of the battery. Only the most relevant S-ISIS spectroscopic images are shown in Fig. 3 for the first cycle; the remaining images are given in supplementary materials along with images of the second cycle (Supplementary Fig. 4). The S-ISIS image of the pristine battery (red dot in the electrochemical curve) is also shown in Fig. 3b.

The ^7^Li signature in the spectroscopic dimension (characterized by its width and shift in the ^7^Li spectrum) is influenced by the paramagnetism of Co and Ti and it is therefore indicative of the degree of lithiation. During charge, the ^7^Li NMR spectrum of LCO broadens and shifts to higher p.p.m. values due to increased paramagnetism and, later, to metallicity of the Li_*x*_CoO_2_ phase in the electrode^44^. Meanwhile, the LTO peak shifts to lower p.p.m. values and broadens, owing to the increased paramagnetism of Ti and the transfer of lithium from tetrahedral 8a to octahedral 16c site as Li_4_Ti_5_O_12_ transforms into Li_7_Ti_5_O_12_ (refs 45, 46). In Fig. 3c, close to half-charge, the NMR spectrum presents the clear signature of an inhomogeneous lithiation process: the region closest to the separator (red arrow) in the LTO electrode is preferentially lithiated (broader and more shifted signal). The LCO electrode also displays a small gradient of lithiation at that stage, although to a lesser extent. Further charging reduces the gradient in lithiation for LTO, and at the end of the charge (*x*=0.54, Fig. 3d) the lithium content inside the two electrodes is more homogeneous. Upon discharge, a small gradient appears again in LTO, as evidenced by the asymmetry of the LTO contour in Fig. 3e, which disappears at the end of discharge. Good reproducibility is observed upon further cycling (Supplementary Fig. 4). After one cycle, the signal from LTO is broader and more intense (faster *T*_*1*_ longitudinal relaxation) than initially observed due to a few paramagnetic Ti^(III)^ that were not oxidized back to Ti^(IV)^.

### Quantification of the gradient of lithiation

**Figure 4:**
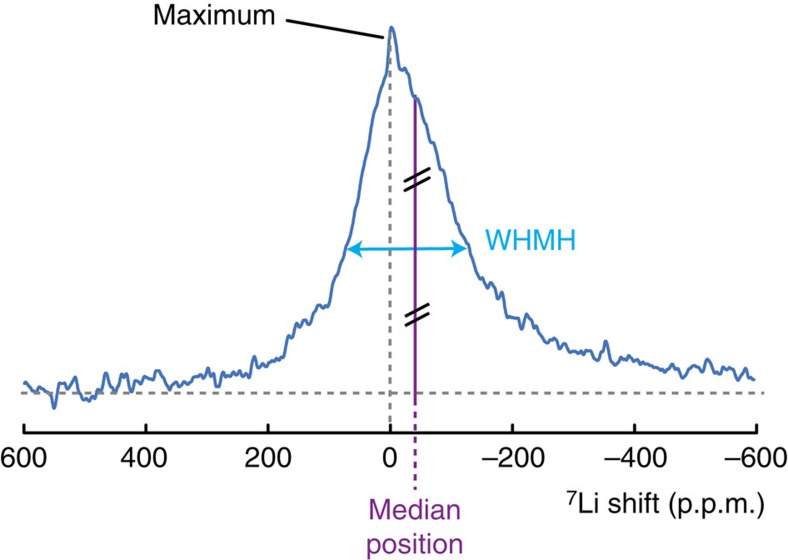
Metrics used to follow the spectral evolution. The median position is defined as the position for which the area on the left side is the same as on the right side. The width at half median height (WHMH) gives a good estimate of the width of the peak, even if it is asymmetric.

**Figure 5:**
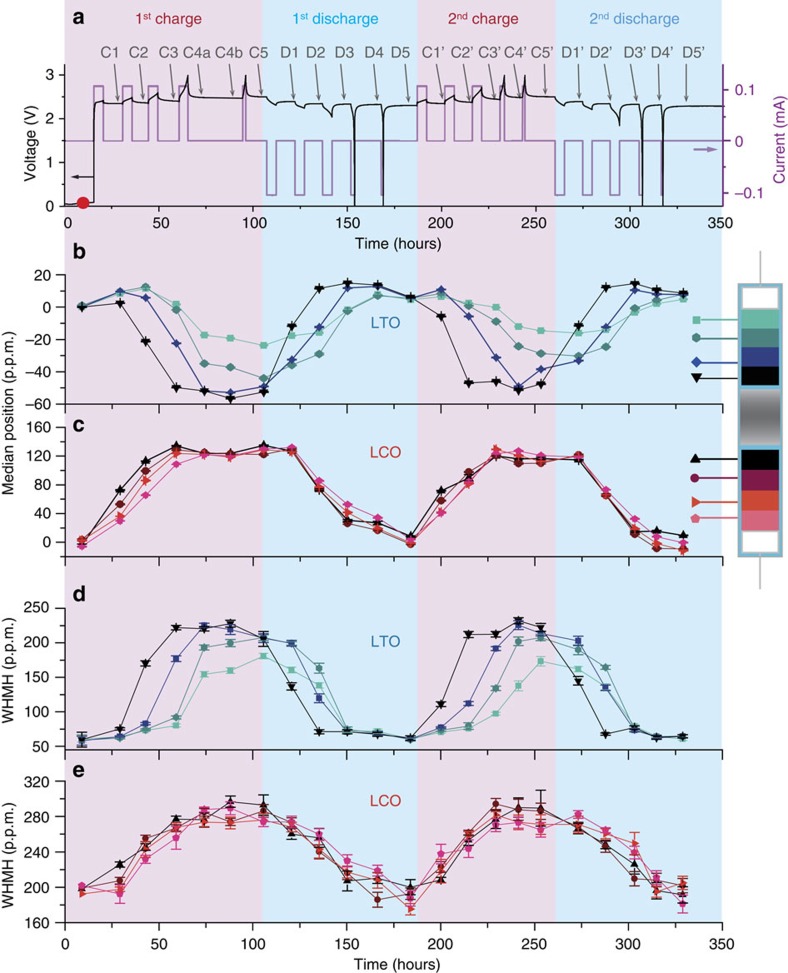
Evolution of the localized *in situ* spectra. (**a**) Voltage and current as a function of time. (**b**,**c**) Evolution of the median position for four 100 μm-thick slices inside the LTO and LCO electrodes. The slices are colour-coded using increasing brightness with the distance from the separator. (**d**,**e**) Evolution of the full width at half-median height (WHMH) for each slice. Error bars indicate the uncertainty from the fit as described in Methods.

To quantify the gradient of lithiation in each electrode upon cycling, we cannot reliably measure the maximum of the electrode NMR spectrum. The maximum is masked by the sharper peak arising from electrolyte soaking all the parts of the electrochemical cell and the complex asymetric lineshape cannot be fitted without additional information (too many unconstrained parameters). We rely instead on the median position and the width at half median height (WHMH) of the spectrum to evaluate the evolution of the broad spectra of the electrodes during battery operation for each slice (Fig. 4). The median is very robust for peak position determination. Note that the median position measured on the asymmetric spectrum in static conditions is different from the peak position measured in magic angle spinning NMR^44^,^46^, most probably due to anisotropic magnetic susceptibility effects. Figure 5 shows the results of such analysis for the *in situ* battery.

In Figure 5, the distribution of median positions and widths across the four 100 μm-thick slices of the negative LTO electrode near half-charge indicates the onset of a macroscopic lithiation gradient, with a preferential lithiation of the region near the separator (darker lines in Fig. 5b,d). Such a gradient is maintained until the end of the first charge in LTO. Upon discharge, the slice near the separator (black line in Fig. 5b,d) is delithiated faster than its counterparts. It is worth noting in each electrode the convergence of the four Li concentration curves towards the end of the first charge-discharge cycle. It indicates a homogeneous distribution of lithium in the electrode and therefore a good reversibility of the Li uptake-removal process. Fig. 5b,d also indicate that this phenomenon is not restricted to the first cycle, since a similar lag in lithiation is found in LTO through the second cycle. Turning to the LCO electrode (Fig. 5c,e), a small lithiation gradient appears at the beginning of the first charge that vanishes in the last stage of the first charge. LCO behaves homogeneously within our experimental resolution (100 μm) upon subsequent cycling, with very similar spectral characteristics for all slices (Fig. 5c,e).

## Discussion

We developed the S-ISIS methodology to provide spectroscopic images for materials with short relaxation times. This approach is limited by two parameters. The first is the longitudinal relaxation time *T*_*1*_; relaxation during the gradient switch reduces the signal intensity and sensitivity can be an issue. The second limiting parameter is the linewidth, as it will impact resolution (details on limitations in Supplementary Discussion). The challenge, therefore, increases with paramagnetism. Preliminary studies indicate that S-ISIS is successful for *T*_*1*_ as short as 2.5 ms at the price of lower sensitivity, and with distortions that can be corrected for.

Fast longitudinal relaxation is also an issue in pure imaging of fast-relaxing solids. Here, relaxation characteristics depend on the extent of lithiation (oxidation state of the paramagnetic center in the electrode). The signal intensity is weighted by relaxation, so that single intensities are not representative of the number of lithium ions in the material. The spectroscopic information, not available in pure MRI, makes the analysis more reliable than for a pure image. An additional asset of this partitioning technique, not performed so far, is the possibility to access single-slice NMR properties (such as relaxation or self-diffusion coefficients) for lithium in electrode materials.

Regarding the battery, the S-ISIS technique allows a detailed characterization of the electrodes. Thanks to S-ISIS, we detect a lithiation front inside LTO, even at the slow cycling rates used here and after letting the battery stabilize significantly at each SOC before the NMR measurement. The LCO electrode behaves more homogeneously. Such results could be surprising based on the known ‘zero-strain' insertion in LTO^47^. The faster lithiation close to the separator points towards ionic transport within the electrode as the limiting factor. Electronic conduction is not an issue in our case, as it would result in faster lithiation near the current collector. Liquid electrolyte penetration inside the electrode is most probably the issue as the electrode is thick and dense, which is critical for diffusion. Here S-ISIS identifies electrode processing as a source of kinetic limitation for LTO, within the actual Li-ion cell configuration.

To conclude, we demonstrate the spectroscopic image of lithium in paramagnetic electrodes inside a full electrochemical cell. Our one-dimensional results may be transferred easily to three-dimensions once the technical challenge of a strong three-dimensional pulsed field gradient has been addressed. Using the S-ISIS methodology, we succeed in monitoring the lithiation distribution through thick electrodes with a 100 μm resolution, inside a battery operating in real conditions. Spatial localization of lithiation fronts in electrodes with S-ISIS provides a non-invasive tool to diagnose limitations in batteries, especially limiting steps in battery performance. For instance, ionic, electronic and more generally transport limitation, related either to the material itself or to the electrode fabrication process, can now be identified based on the combination of spatial and spectroscopic information. We observe inhomogeneous lithiation for thick electrodes, even made from materials known for their good cycling behaviour in thin electrodes. These results call for special attention regarding thick electrode processing, which appears crucial even for batteries made from materials with fast-cycling capability. On a broader scope, the ISIS method can be extended to a variety of fast-relaxing solids to map their chemical composition, by taking advantage of the richness of NMR-active elements such as ^1^H, ^11^B, ^19^F, ^23^Na, ^27^Al, ^31^P, ^67^Zn... Along this line, this non-destructive spatially resolved characterization method could be of great help for deciphering chemical inhomogeneity in materials containing paramagnetic ions, such as precious rocks or glass. It further paves the way to study, in real time, each constituent in devices with paramagnetic materials, among which batteries, fuel cells or reactors involving solid-driven catalytic, crystallization or vitrification processes.

## Methods

### Active material

Nanosized lithium titanate spinel oxide (Li_4_Ti_5_O_12_, particle size<100 nm) and lithium cobalt oxide (LiCoO_2_) were purchased from Aldrich. The magic angle spinning ^7^Li NMR spectrum of LiCoO_2_ reveals a small amount of paramagnetic defects, indicating a slight overstoechiometry (*x*∼1.05) by comparison with ref. 48.

### Battery assembly

The active material (Li_4_Ti_5_O_12_ or LiCoO_2_) was mixed with polyvinylidene fluoride (Kynar Flex 2801 from Arkema) and carbon super P (Timcal) (80:10:10 w/w/w) in acetone in an agate mortar. Dense electrode pellets (5 mm-diameter) were pressed with a uniaxial press at 2 tons and dried at 120 °C for 24 h under vacuum before assembly in the glove box. SEM micrographs are shown in Supplementary Fig. 1.

The battery was assembled in a Jacomex argon-filled glove box, using the electrochemical cell that we developed previously for *in situ* NMR measurements^25^. The 444 μm-thick positive electrode had a theoretical surface capacity of 13.46 mAh cm^−2^ considering 0.5 Li per Co (24.1 mg). The 495 μm-thick negative electrode had a theoretical surface capacity of 14.48 mAh cm^−2^ considering 3 Li per Ti (20.3 mg). The two electrodes were separated by two pieces of porous glass microfiber (Whatman, type GF/D). The whole content of the battery was soaked in LP30 electrolyte (1 mol l^−1^ LiPF_6_ in ethylene carbonate and dimethyl carbonate in weight ratio 1:1, Merck). The current collectors were made of aluminium and copper.

### Galvanostatic cycling

Galvanostatic cycling (Supplementary Fig. 3) was carried out at a current of 115 μA (theoretically 0.5 Li per LiCoO_2_ unit in 25 h, C/25), using a Biologic VSP galvanostat. The current was paused five times (open circuit) during charge or discharge to prevent evolution during acquisition of the S-ISIS spectroscopic images. Each open-circuit stage started with 7 h to let the battery stabilize before recording the S-ISIS data. In all cases, the voltage stability was such that it changed by <3 mV in an hour. The first charge was paused at *x* values (in Li_*x*_CoO_2_) of 0.89, 0.78, 0.68, 0.58, 0.54. The first discharge was also paused five times: *x*=0.65, 0.75, 0.86, 0.91 and 0.93. The electrochemical data indicate that 0.93 Li per Co are back in the first cycle. For the second cycle the pauses were for *x*=0.83, 0.72, 0.61, 0.56, 0.53 (second charge) and 0.64, 0.75, 0.85, 0.89 and 0.92 (second discharge). The charge was stopped when the voltage reached 3 V (chosen as a safety to prevent charging further than Li_0.5_CoO_2_). The discharge was stopped when the voltage reached 0 V. Further charging or discharging was attempted one time after reaching the voltage limit and relaxing for each charge/discharge. The results obtained with the *in situ* cell were validated by comparison with coin cells made with the same protocol (Supplementary Fig. 5).

### Scanning image-selected *in situ* spectroscopy

Details of the ISIS approach are provided in Supplementary Discussion and Supplementary Fig 6, 7 and 8. The NMR and electrochemistry were synchronized using TTL pulses. All MRI and NMR spectroscopy measurements were carried out on a Bruker Avance III HD spectrometer operating at 77.7 MHz (^7^Li Larmor frequency at 4.7 T) equipped with a Bruker diffusion probe with a 0.5 T m^−1^ A^−1^ vertical gradient, and a 60 A Bruker Great60 pulsed field gradient amplifier. We acquired 17 slices of thickness 100 μm, with no slice gap. Sixteen dummy scans were performed at the start to reach a stationary state, and 512 transients were recorded for each slice. The field of view was 1.7 mm, larger than the LCO/electrolyte/LTO portion (1.3 mm). The experimental time to obtain 17 slices and a reference spectrum was about 2 h 45 min, except for the pristine battery (see below). A spectral width of 200 kHz and an acquisition time of 10.3 ms were used in the spectroscopic dimension. Hard pulses were performed with a radio-frequency (RF)-field of 20 kHz (50 W), and a PFG of 23.968 Tm^−1^ was used for spatial selection. Spatial selection of a slice under PFG was performed with a selective inversion pulse. The offset of the selective pulse is varied to observe a given slice. We used IBURP1 (ref. 49) and Hyperbolic Secant^50^,^51^ inversion pulses, set so that the inversion bandwidth is slightly <40 kHz (duration 112.4 μs and peak RF power of 120 W for IBURP1, duration 500 μs and peak RF power of 20 W for Hyperbolic Secant).

The S-ISIS image of the pristine battery was obtained in the same conditions except for a longer repetition time of 10 s to allow partial relaxation of LTO (longitudinal relaxation rate *T*_*1*_ of 10 s) and full relaxation of LCO (longitudinal relaxation time *T*_*1*_ of 0.5 s). Only 64 transients were acquired for each slice to keep acquisition time reasonable (3 h 20 min). For all the other ISIS data, the repetition times were set between 1 and 2 s. The longitudinal relaxation rates *T*_*1*_ of the electrodes during cycling ranged from 290 ms (end of charge) to 500 ms (full discharge) for the LCO electrode and from 150 ms (end of charge) to 350 ms (full discharge) for the LTO electrode.

### Chemical shift imaging

For comparison, the CSI image of the pristine battery was obtained in 3 h 38 min using a standard spin-echo CSI sequence with 64 scans and a recycle time of 10 s. The image dimension was acquired in 40 increments using a rectangular gradient pulse of duration 150 μs, a maximum strength of 3.16 T m^−1^ and a gradient stabilization time of 200 μs, chosen to avoid distortion of the image. Using a stronger gradient strength necessitates a longer gradient stabilization time before the RF pulse (to prevent distortion of the image), which would exclude detection of the paramagnetic electrodes due to their fast transverse relaxation *T*_*2*_'. Even such short stabilization times do not allow detection of the paramagnetic electrodes. The spectrum was processed with an exponential apodization in the direct dimension (50 Hz) and no apodization in the indirect dimension. The field of view was 2.5 mm with a resolution of 77 μm.

### Scanning image-selected *in situ* spectroscopy data analysis

All spectra were processed with the same parameters. The signal was apodized with an exponential before Fourier transformation (500 Hz). The S-ISIS spectroscopic image was reconstructed with a home-made script (AU program) in Topspin. Chemical shifts were referenced to the most intense peak in the electrolyte at 0 p.p.m. The S-ISIS figures shown in the paper were calibrated in the spatial dimension with the dmfit software^52^.

The median is defined as the position for which the area of the peak on the left and on the right are equal. These measurements were automated using Matlab (The MathWorks, Inc.). We used S. Cadars's NMR processing scripts to read the data in Matlab (part of the functions are from MatNMR^53^) and we wrote a home-made function to determine automatically the median position and WHMH.

Errors in the fit were determined as follows: for each ISIS slice at each SOC, 50 data sets were generated by adding noise from a normal distribution with a variance set to the level of noise measured on the spectra. The median position was determined on these data sets. The standard deviation in the set of 50 fits was calculated for each ISIS slice and it was then used for the error bars. The same procedure was applied to determine the errors in the fit for the WHMH.

### Data availability

The data that support the findings of this study are available from the corresponding authors upon request.

## Additional information

**How to cite this article:** Tang, M. *et al*. Following lithiation fronts in paramagnetic electrodes with *in situ* magnetic resonance spectroscopic imaging. *Nat. Commun.* **7,** 13284 doi: 10.1038/ncomms13284 (2016).

**Publisher's note:** Springer Nature remains neutral with regard to jurisdictional claims in published maps and institutional affiliations.

## Supplementary Material

Supplementary Information Supplementary Figures 1-8 and Supplementary Discussion
