# Supplementary material for: Following lithiation fronts in paramagnetic electrodes with in situ magnetic resonance spectroscopic imaging
Source: Nat Commun. 2016 Nov 3;7:13284. doi: 10.1038/ncomms13284 (PMC5097146; doi:10.1038/ncomms13284)
Supplement: Supplementary Information — Supplementary Figures 1-8 and Supplementary Discussion [file ncomms13284-s1.pdf]

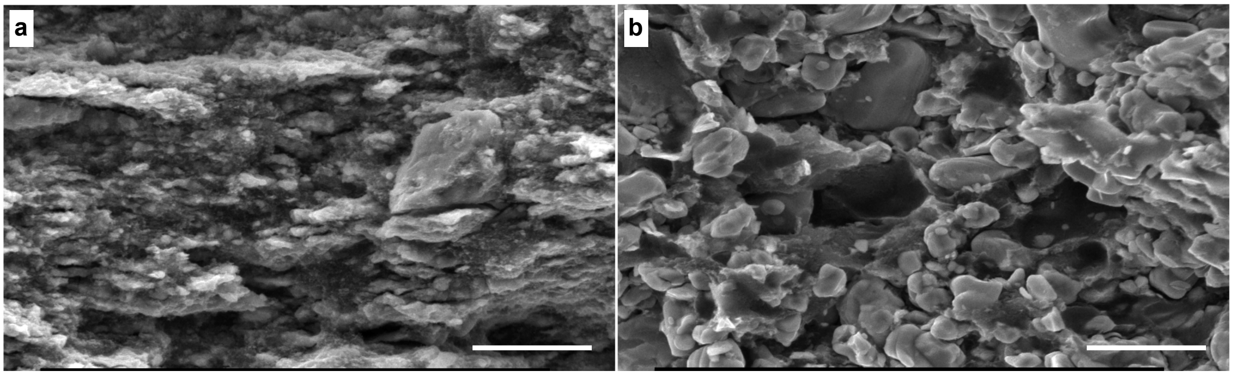

Supplementary Figure 1. Scanning Electron Microscopy images of the pristine electrodes. (a) negative electrode and (b) positive electrode. Images were performed using a FEI/Philips XL40 microscope with a secondary electron detector and an accelerating voltage of 20 kV. Scale bars: 20 µm.

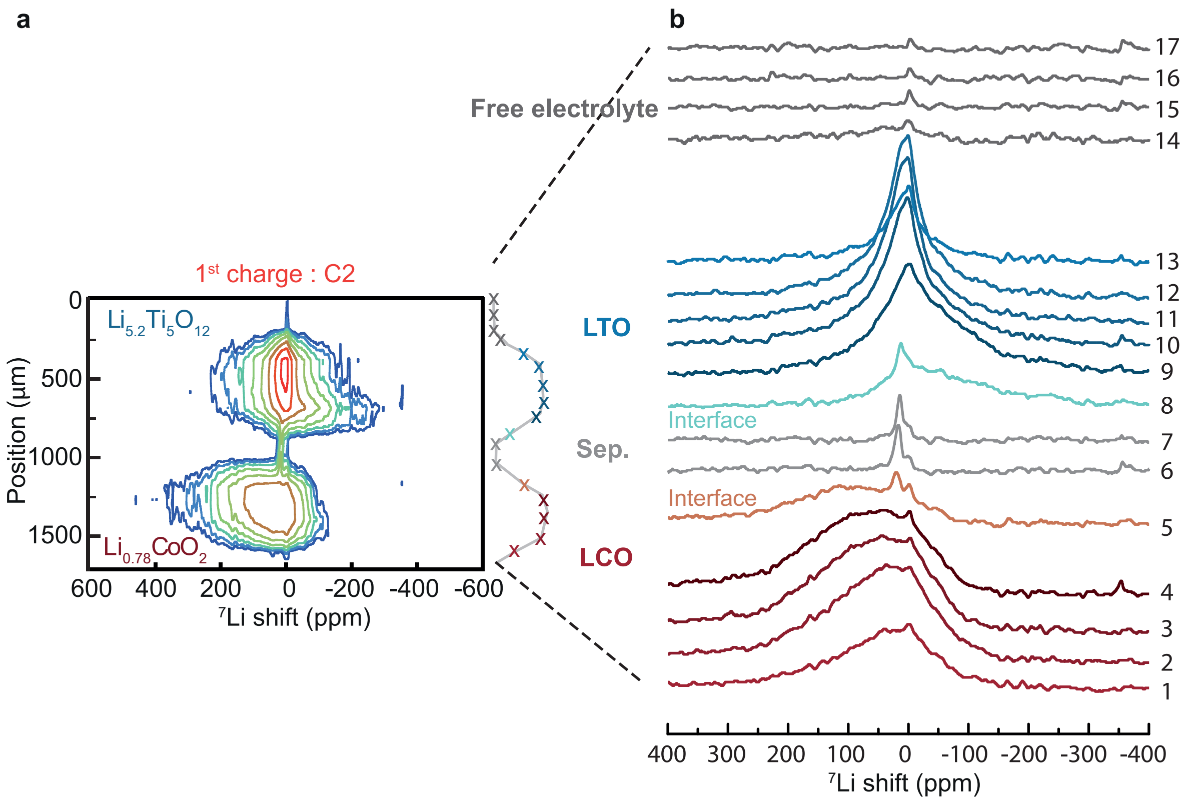

Supplementary Figure 2. LCO/LTO battery charged for 10 hours. (a) ^{7}Li Scanning ISIS spectroscopic image (slice thickness 100 μm). (b) Series of spectra extracted from the S-ISIS spectroscopic image. The liquid electrolyte signal is small due to partial saturation of its NMR signal. Slices 5 and 8 are less intense because they are astride the interface of electrode and the separator.

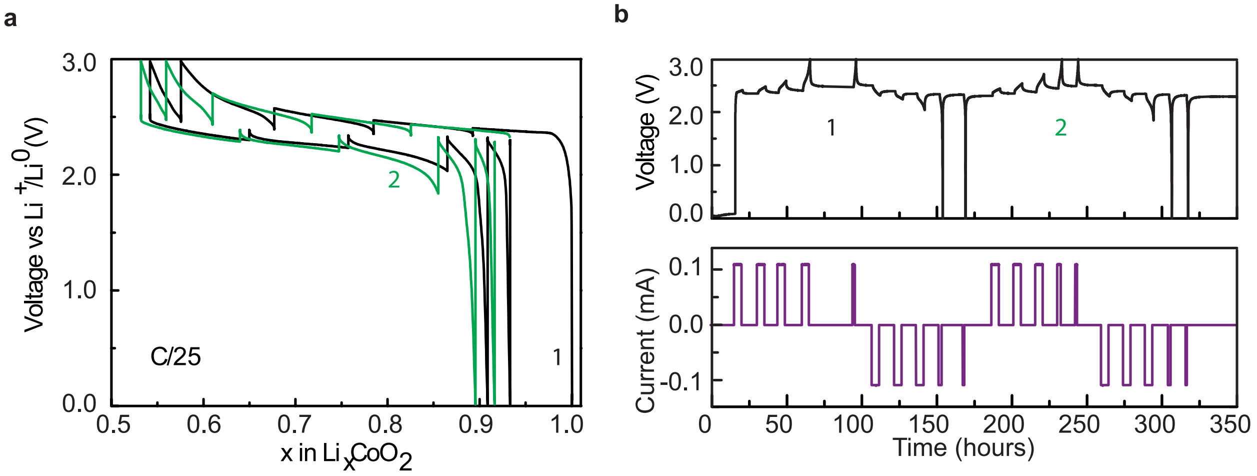

Supplementary Figure 3. Electrochemical characteristics of the *in situ* battery. (a) Working voltage as a function of x in Li_xCoO_2. The cycle number is indicated close to the curve. (b) Working voltage and (c) current as a function of time.

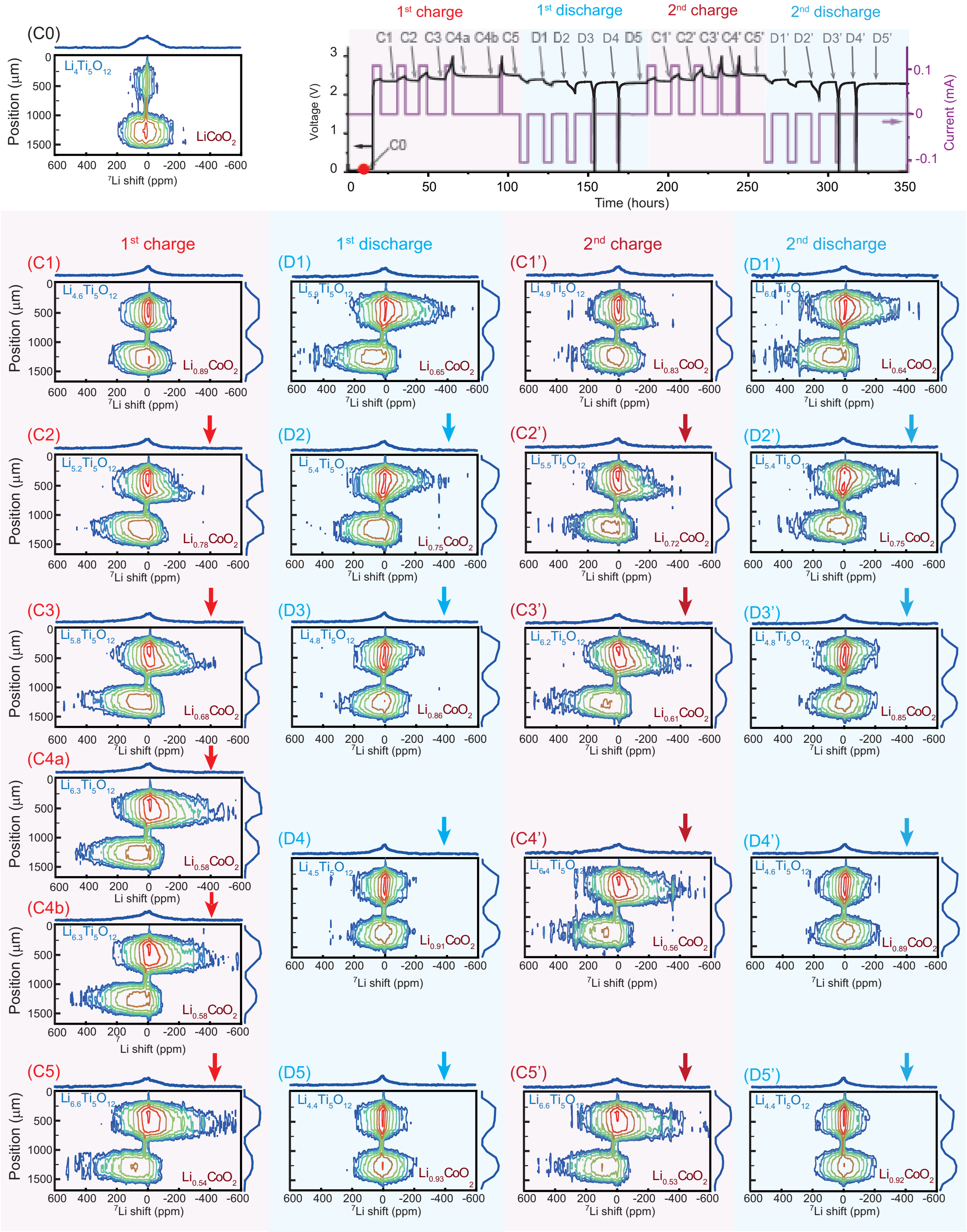

Supplementary Figure 4. S-ISIS images of the first two cycles. All images were recorded after letting the battery relax at the given lithiation stage in open-circuit mode as shown in the electrochemical profile. The spectroscopic images in the first and second charge (first and third columns) are very similar and indicate good behaviour of the battery. This is also true in the first and second discharges (second and fourth columns).

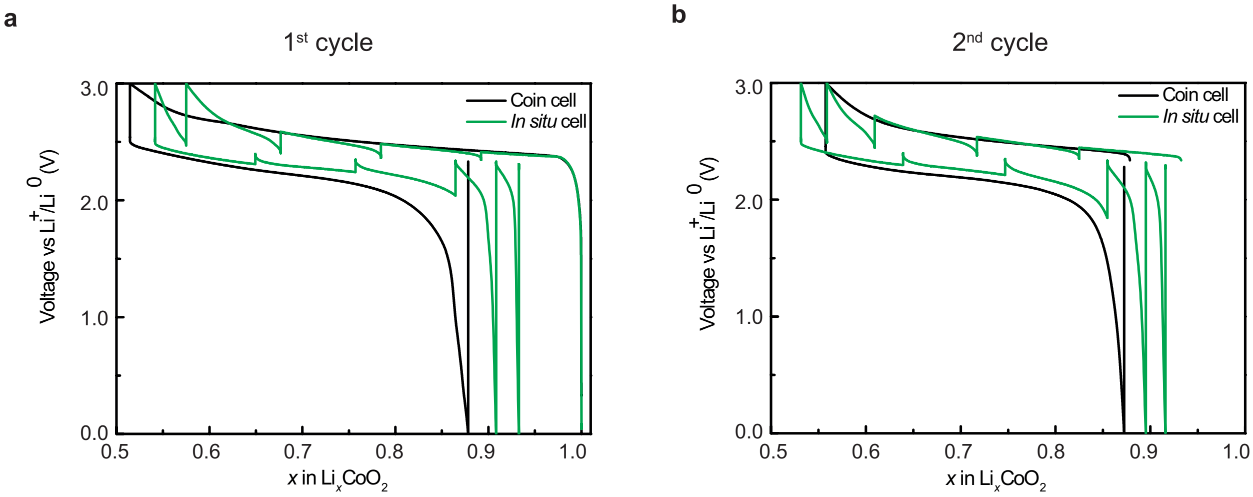

Supplementary Figure 5. Comparison of the electrochemical profiles of a coin cell and the *in situ* cell. (a) First cycle and (b) Second cycle at C/25. Electrodes of the coin cell are similar to those used in the *in situ* battery (thickness 515 µm for LTO, 461 µm for LCO). The cycling conditions were identical except for the open-circuit stages for S-ISIS imaging in the *in situ* battery, which created the vertical lines in the electrochemical profile of the *in situ* battery (no change in x but voltage relaxation).

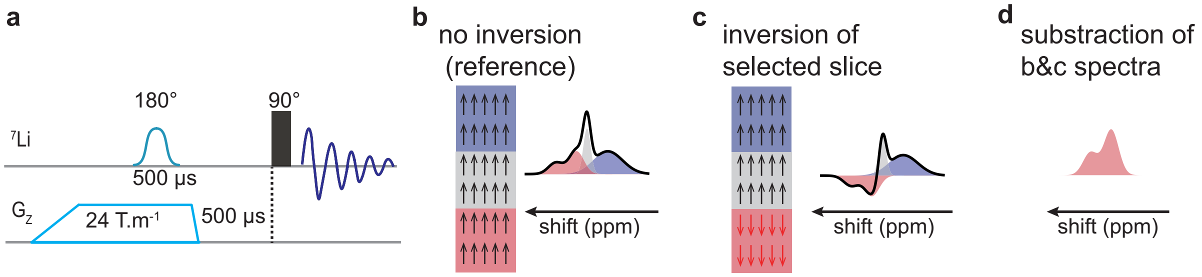

Supplementary Figure 6. Acquisition of a localized spectrum with ISIS. (a) ISIS pulse sequence. (b) Schematics of the battery without inversion pulse and resulting reference spectrum. (c) Schematic of the battery with a selective inversion pulse targeting the bottom slice and resulting spectrum. (d) Spectrum of the selected slice obtained from the difference of the spectra in b and c.

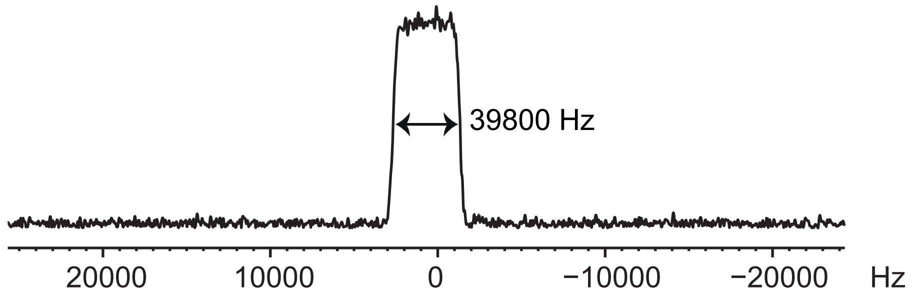

Supplementary Figure 7. Profile of the selective Hyperbolic secant pulse. It was obtained with a selective echo under gradient in a solution of lithium chloride in a vertical tube (gradient of 3 T m^{-1}). Hyperbolic secant pulse duration: 500 µs, power: 20 W.

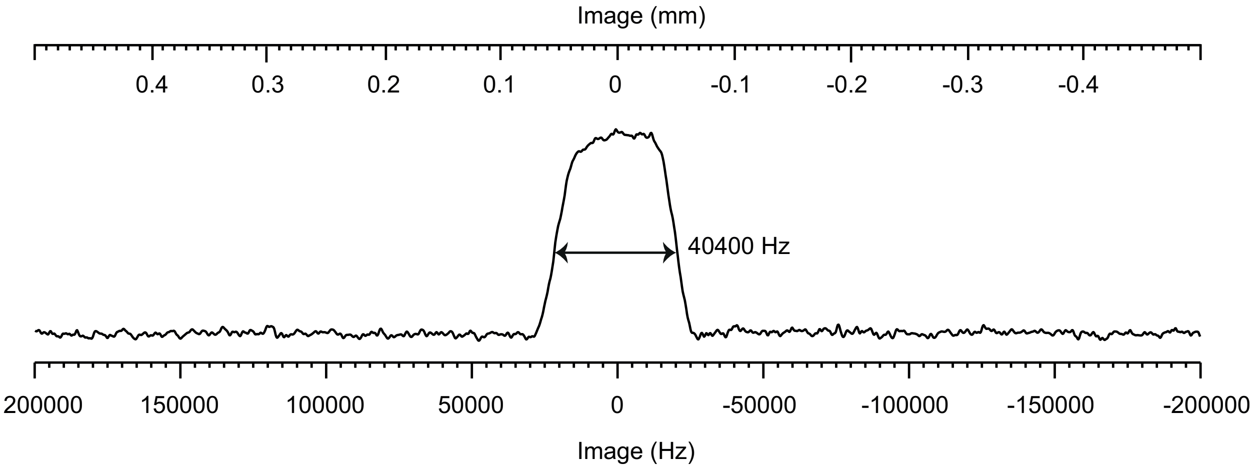

Supplementary Figure 8. Profile of the slice selected in a tube containing 1 cm of LCO. The profile is obtained by subtraction of a reference spectrum to reproduce the ISIS approach. We used the same selective pulse and gradient strength as for the *in situ* study.

Supplementary Discussion

The concept behind ISIS (Image Selected *In situ* Spectroscopy) is to take advantage of the slightly longer longitudinal relaxation time and to obtain the localized spectrum of a specific slice within the sample. In Scanning ISIS (S-ISIS) the full image is reconstructed by combining all the slices.

Acquisition of a localized spectrum

Two experiments are required for acquiring a slice with ISIS. The first experiment inverts the spins in the slice of interest. A subsequent 90° radio-frequency pulse creates transverse coherence for detection (Supplementary Fig. 6), with negative contribution from the inverted spins in the slice of interest (red) and positive contribution from the unperturbed spins (grey and blue) of the sample. A second experiment acquires a spectrum of all ^{7}Li nuclear spins within the field of view (along the vertical direction in this study) as a reference. Subtraction between both spectra cancels out the signal from the unperturbed spins while it increases the signal of the selected slice.

The inversion of the slice of interest is performed with a selective pulse. The offset of the selective pulse controls the position of the slice while the range of frequencies excited by the selective pulse sets the thickness of the slice. Supplementary Fig. 7 shows the profile of the Hyperbolic Secant pulse used in ISIS on a solution of lithium chloride. The width at half height is 39800 Hz.

Slice thickness and resolution

The thickness of the slice is set by the range of frequencies excited by the selective pulse.

$$\Delta z = {}^{\Delta\nu_{\mathrm{imag}}}\!\big/_{\gamma G_{\mathrm{z}}}$$

There are two possibilities: the bandwidth of the selective pulse ($\Delta\nu_{\mathrm{p}}$) is set (1) smaller or (2) broader than the linewidth of the peak under no gradient ($\Delta\nu_0$) .

Case (1): $\Delta\nu_{\mathrm{p}} < \Delta\nu_0$, $\Delta\nu_{\mathrm{imag}} = \Delta\nu_0$. The slice thickness varies across the battery and changes with the state of charge.

Case (2): $\Delta\nu_{\mathrm{p}} > \Delta\nu_0$, $\Delta\nu_{\mathrm{imag}} = \Delta\nu_{\mathrm{p}}$. The thickness is constant and equal to $\Delta\nu_{\mathrm{p}}$, with a displacement of the pixel depending on the chemical shift.

We set up the experiment so as to respect case (2) for all slices and during the whole process of charging/discharging the battery. We used $\Delta\nu_{\mathrm{p}}$=40 kHz, corresponding to a thickness of 100 µm (G_z=23.968 T m^{-1}, γ=16.55·10^{6} Hz T^{-1}).

Scanning ISIS (S-ISIS)

In Scanning-ISIS, the ISIS procedure is repeated while varying the offset of the selective pulse so as to cover all the slices in the sample. The reconstruction of the full spectroscopic image is performed by concatenating the spectra of all the slices. The S-ISIS image at half charge C2 is shown in Supplementary Fig. 2 with the corresponding spectra for each slice.

Limitations

The slice selected with ISIS is displaced as a function of the offset frequency; the width of the spectrum under no gradient $\Delta\nu_0$ creates a blurring.

Supplementary Fig. 8 shows the image of the 100 μm-thick slice (40.4 kHz) selected in a tube containing 1 cm of powdered LCO ($\Delta\nu_0$=13 kHz at half maximum) with the same selective pulse and gradient strength as in S-ISIS on the *in situ* battery. It was obtained with subtraction of a reference spectrum to reproduce the ISIS approach. The blurring is expected to be around 7.5 kHz (19 μm) on each side. Interestingly, the slice thickness is only broadened by 600 Hz (2 μm), so that blurring can be considered negligible.

In the battery during charge and discharge the worst case is encountered at full charge for LTO, with $\Delta\nu_0 \approx 30$ kHz. As a consequence, a blurring of 38 μm is expected on each side of the pixel, which should result in an increase of the apparent thickness of the LTO electrode. We do not observe such effect in the contour plots of Fig. 3 and Supplementary Fig. 4, and we consider that this effect is negligible in our case.

Another potential source of blurring is lithium diffusion after the gradient pulse. It is negligible for solid electrodes as self-diffusion coefficients in such solids are at best 10^{-11} m^2 s^{-1}. Lithium diffusion in the electrodes would result in a blurring of the slice thickness of less than 0.1 μm given our gradient stabilization time (500 μs).
